# Artificial Intelligence-Enhanced Telerehabilitation in Post-Acute Coronary Syndrome: A Narrative Review of Opportunities, Evidence, and Future Directions

**DOI:** 10.3390/life16030444

**Published:** 2026-03-09

**Authors:** Alina Gherghin, Mircea Ioan Alexandru Bistriceanu, Ilie Onu, Daniel Andrei Iordan, Florentin Dimofte, Adriana Neofit, Dan Eugen Costin, Alexandru Scafa-Udriste

**Affiliations:** 1 Doctoral School, “Carol Davila” University of Medicine and Pharmacy, 050474 Bucharest, Romania; alina-claudia.mortoiu-vasinca@drd.umfcd.ro (A.G.); mircea-ioan-alexandru.bistriceanu0721@stud.umfcd.ro (M.I.A.B.); alexscafa@yahoo.com (A.S.-U.); 2 Emergency Clinical Hospital, 014461 Bucharest, Romania; 3 Department of Biomedical Sciences, Grigore T. Popa University of Medicine and Pharmacy Iasi, 700454 Iasi, Romania; 4 Center of Physical Therapy, Rehabilitation and Wellness, “Dunărea de Jos” University of Galati, 800008 Galati, Romania; florentin.dimofte@ugal.ro (F.D.); adriana.neofit@ugal.ro (A.N.); dan.costin@ugal.ro (D.E.C.); 5 Department of Individual Sports and Kinetotherapy, Faculty of Physical Education and Sport, “Dunărea de Jos” University of Galati, 800008 Galati, Romania; 6 Surgical Department, Faculty of Medicine and Pharmacy, “Dunărea de Jos” University of Galati, 800008 Galati, Romania

**Keywords:** artificial intelligence, telerehabilitation, acute coronary syndrome, exercise

## Abstract

Cardiac telerehabilitation has become a promising alternative to traditional programmes for preventing acute coronary syndrome (ACS) in the secondary phase. However, current implementations are still reactive and standardised, lacking personalisation and flexibility in clinical settings. By integrating artificial intelligence (AI), it may be possible to overcome these limitations and provide intelligent, scalable, and patient-centred care. Methods: We conducted a structured literature review across PubMed, Scopus, the Cochrane Library, and Web of Science, targeting English-language studies published from January 2015 to May 2025. Inclusion criteria included adult populations with a history of ACS or high cardiovascular risk, assessing interventions based on AI, telerehabilitation, or their combination. Studies are needed to report clinical, functional, behavioural, or technological outcomes. A thematic narrative synthesis was utilised. Results: AI-enhanced telerehabilitation demonstrates potential advantages over conventional digital care in selected domains, including adaptive risk prediction, personalised exercise modulation, and adherence support. Several systems report real-time adjustment of exercise protocols, early dropout detection, and predictive analytics for rehospitalisation. AI integration may also contribute to personalised behavioural feedback and psychosocial monitoring. Nevertheless, the overall level of evidence remains preliminary and heterogeneous, with most AI-based interventions evaluated in pilot, feasibility, or modelling studies rather than large-scale randomized trials. Conclusions: The integration of AI into telerehabilitation represents a promising evolution in post-ACS care, shifting from predominantly reactive monitoring toward more adaptive and data-driven support models. While early-phase studies suggest feasibility and potential clinical benefit, robust multicentre randomized controlled trials and cost-effectiveness analyses are required before definitive conclusions regarding superiority or widespread implementation can be drawn.

## 1. Introduction

Cardiovascular diseases remain the leading cause of death and illness worldwide, with acute coronary syndrome (ACS) being a critical stage associated with a high risk of recurrence and subsequent deterioration. In this context, secondary prevention becomes a top priority. However, conventional cardiac rehabilitation (CR) is often limited by restricted accessibility, high costs, and low patient compliance.

Over the past two decades, the use of digital technologies to deliver remote rehabilitation programmes has emerged as a viable and effective alternative to traditional CR. Studies have shown this approach can significantly increase patient participation and reduce post-infarction adverse events. Nevertheless, in its current form, telerehabilitation remains largely standardised and reactive, lacking real-time personalisation, dynamic adaptability, and proactive clinical risk prediction.

Against this background, the integration of artificial intelligence (AI) into telerehabilitation is emerging as an innovation and a necessity in modern cardiovascular medicine. AI—encompassing machine learning (ML) and deep learning (DL) algorithms—has already been successfully applied across multiple domains in cardiology, including ECG interpretation, risk stratification, event prediction, and treatment personalisation [1].

A meta-analysis by Krittanawong et al., involving over 3 million patients, demonstrated that ML algorithms such as boosting and support vector machines (SVMs) achieved high predictive accuracy (AUC 0.88–0.93) for coronary artery disease and stroke [2]. Similarly, Hill et al. showed that AI can detect arrhythmias such as atrial fibrillation using routine clinical data, providing a valuable pathway for active monitoring, particularly during the vulnerable post-ACS period [3].

AI has the potential to go beyond early detection and reshape therapeutic approaches by enabling personalised telerehabilitation. Krittanawong et al. highlighted AI’s ability to create individualised risk profiles, allowing for tailored session intensity, frequency, and content [4]. Furthermore, Golas et al. developed a machine learning model using electronic health records to predict the risk of 30-day rehospitalisation in heart failure—an approach that could be easily applied to the post-infarction context [5]. Early proteomics-based studies, such as the one led by Rossing et al., also suggest that AI-driven biomarker analysis could improve risk stratification and adjustment of interventions [6].

Although AI and telerehabilitation have made significant progress, they have mainly been used as separate tools in cardiovascular care, with little research exploring how they work together within a combined clinical framework. A recent review of European clinical research found that most AI-related projects focus on diagnostics or technological feasibility, while AI-assisted behavioural interventions and telerehabilitation are under-researched through robust randomised trials. Furthermore, the first two weeks after an ACS—a crucial period for relapse, non-adherence, and hemodynamic instability—are under-represented in current research, despite being the time when AI could have the greatest impact through rapid triage, decompensation prediction, and dynamic rehabilitation adjustment. Patients with unstable angina (UA) and non-ST elevation myocardial infarction (NSTEMI) are also often excluded from standard protocols, even though AI-based tools are already showing promise in differential diagnosis and early risk stratification [7].

Consequently, this narrative review seeks to fill these gaps through a critical and integrative analysis of the existing literature on the combined effect of AI and telerehabilitation in cardiovascular prevention. The emphasis will be on the subacute post-ACS phase, the role of AI in personalising rehabilitation, managing risk factors, user experience, and emerging applications of intelligent digital health technologies.

## 2. Materials and Methods

This work was designed as a structured narrative review, chosen deliberately to address the rapidly evolving and highly heterogeneous field of artificial intelligence (AI)–enhanced cardiac telerehabilitation in the post-acute coronary syndrome (ACS) setting. Given the diversity of intervention designs, AI methodologies, outcome measures, and implementation stages, a narrative synthesis was considered more appropriate than a formal systematic review or meta-analysis, which would have limited the ability to capture emerging clinical applications, feasibility studies, and translational technologies.

### 2.1. Literature Search Strategy

A comprehensive literature search was conducted across four major electronic databases: PubMed, Scopus, the Cochrane Library, and Web of Science. The search was restricted to English-language publications released between January 2015 and May 2025, a period corresponding to the clinical maturation of machine learning and deep learning models, wearable-integrated systems, and scalable mobile health (mHealth) platforms in cardiovascular rehabilitation.

The search strategy combined controlled vocabulary and free-text terms related to telerehabilitation and artificial intelligence, including: “telerehabilitation”, “remote cardiac rehabilitation”, “artificial intelligence”, “AI”, “machine learning”, “deep learning”, “digital health”, in conjunction with cardiovascular terms such as “acute coronary syndrome”, “STEMI”, “NSTEMI”, “unstable angina”, “high-risk ischemic patients”, “secondary prevention”, and “cardiac prevention”. Reference lists of relevant reviews and key articles were also screened to identify additional eligible studies.

For the purpose of this review, AI-based interventions were defined as systems incorporating machine learning or deep learning algorithms capable of data-driven adaptation, prediction, or automated decision support, rather than static, rule-based, or reminder-only digital platforms.

### 2.2. Eligibility Criteria

**Figure 1:**
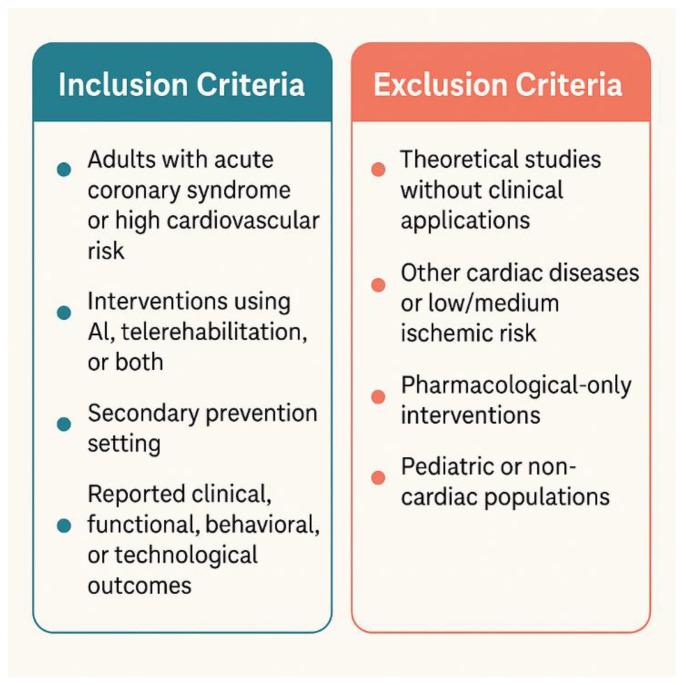
Inclusion and exclusion criteria of the population and intervention.

Eligible studies included adult populations (≥18 years) with a documented history of ACS (ST-elevation myocardial infarction, non-ST-elevation myocardial infarction, or unstable angina) who were enrolled in cardiac rehabilitation or telerehabilitation programmes, with or without AI integration (Figure 1). Studies were required to evaluate AI-based interventions, telerehabilitation, or a combination of both, and to report outcomes related to clinical performance, functional capacity, behavioural change, psychosocial parameters, or technological feasibility.

Included publication types comprised original research articles, randomized or non-randomized clinical studies, feasibility and pilot trials, meta-analyses, clinical guidelines, and narrative or systematic reviews that described validated clinical applications or functional prototypes. Given the early translational stage of AI-enhanced telerehabilitation, feasibility studies and validated technological prototypes were intentionally included to capture implementation potential beyond classical efficacy outcomes.

Exclusion criteria consisted of purely theoretical or conceptual studies without clinical application, studies focused on unrelated cardiovascular conditions (e.g., chronic heart failure, myocarditis), interventions lacking a digital or remote component, pharmacological-only protocols, and studies involving non-cardiac or paediatric populations.

### 2.3. Study Selection and Data Synthesis

Study selection followed a multi-stage screening process. After duplicate removal using reference management software, titles and abstracts were independently screened by two reviewers. Full-text articles meeting the eligibility criteria were subsequently assessed for inclusion. Discrepancies were resolved through discussion and consensus.

A thematic narrative synthesis was performed, with studies grouped according to intervention type (telerehabilitation alone versus AI-enhanced telerehabilitation) and primary outcome domains (clinical, functional, behavioural, psychosocial, or technological). The synthesis focused on identifying recurrent thematic patterns, clinical benefits, implementation barriers, current limitations, and knowledge gaps, as well as emerging opportunities for integrating AI into post-ACS telerehabilitation pathways.

Formal quantitative pooling, comparative effectiveness analysis, and structured risk-of-bias scoring were not undertaken, as these approaches were considered inappropriate given the heterogeneity of study designs, AI models, intervention intensities, and outcome measures.

Figure 1 provides a structured overview of the eligibility framework applied in this narrative review, clearly delineating the clinical scope, intervention criteria (AI, telerehabilitation, or combined approaches), and outcome requirements. The exclusion criteria emphasise the focus on clinically applicable digital interventions and avoid conflation with purely theoretical, pharmacological, or non-cardiac studies. This conceptual boundary was essential to maintain coherence in a field characterised by overlapping AI and cardiovascular research domains.

## 3. Results

A total of 37 original and interventional studies were included in the narrative synthesis (excluding background reviews and conceptual papers). Of these, 18 were randomized controlled trials evaluating telerehabilitation interventions without AI integration, 3 were pilot or feasibility trials, and 16 were AI-focused studies primarily involving predictive modelling, retrospective analyses, or early-phase implementation research. Only a limited number of studies (*n* = 2–3) evaluated fully integrated AI-enhanced telerehabilitation platforms in clinical settings. This distribution reflects a mature evidence base for conventional telerehabilitation but an emerging and predominantly translational evidence landscape for AI-enhanced rehabilitation.

A. Only Telerehabilitation Interventions

Many recent studies have shown that telemedicine interventions can be partially effective in post-infarction cardiac rehabilitation. Text messaging and mobile app-based platforms, as discussed by Santo et al., Zheng et al., Yu et al., Khonsari et al., and Bae et al., have focused on changing patients’ behaviours, especially those related to medication adherence and lifestyle changes. For instance, sending daily or weekly text messages significantly improved completion rates for CR and medication adherence. However, these effects often wore off quickly after the intervention stopped, as they lacked an adaptive mechanism [8,9,10,11,12].

Despite these promising results, the long-term sustainability of telerehabilitation interventions remains limited. User retention, engagement, and outcome maintenance tend to decline progressively after the first 6–12 weeks, as reported by Pfaeffli Dale et al. and Volpp et al. [13,14]. Marvel et al., through the MiCORE programme, documented excellent initial completion rates; however, the gradual decline in patient engagement highlighted the need for dynamic solutions that sustain attention and motivation over time [15].

Interventions combining digital education with physiological telemonitoring have demonstrated moderate benefits; however, these remain reactive and cannot adapt to individual patient profiles. Studies by Treskes et al., Hilu et al., and Dalli Peydró et al. demonstrated that telemonitoring combined with periodic counselling can reduce hospitalisations and enhance patient satisfaction. Nonetheless, these models still rely on manual data interpretation and clinician involvement, which restricts their scalability and efficiency, particularly in overburdened healthcare systems [16,17,18].

One key area of research has compared traditional telerehabilitation with conventional centre-based CR. Studies by Frederix et al., Avila et al., Claes et al., and Kraal et al. have shown that the two approaches are functionally equivalent regarding VO_2_ peak and quality of life. However, these mobile health interventions have not yet matched the outcomes of specialist rehabilitation centres, mainly due to the lack of personalised feedback mechanisms or automatic adjustments based on patient status [19].

Overall, research in this area shows that non-AI digital telerehabilitation is generally feasible and has some effectiveness, but also highlights its limitations. Without an adaptive component to interpret real-time data and tailor interventions, the impact of these programmes plateaus. Additionally, they rely on ongoing human input for data analysis, alert generation, and behavioural counselling, which are becoming increasingly unsustainable in overburdened healthcare systems. These findings stress the pressing need to integrate AI to overcome these constraints and make telerehabilitation a more sustainable, scalable, and personalised intervention [20,21].One major limitation of many of these interventions is that they are rigid and non-adaptive. Non-AI mHealth platforms are based on fixed rules, set protocols, and generalised messages. This means they struggle to address the complexity of modern cardiac patients, who often have multiple health issues, psychological barriers, and changing behaviours. As a result, even when delivered well, these interventions often only help a small group of digitally savvy and highly motivated patients [22,23].

B. AI-Enhanced Telerehabilitation Interventions

Research has shown that incorporating AI into telerehabilitation programmes can benefit significantly, including more personalised interventions, better adherence, and improved functional outcomes. A notable example is the IERMS system, described by Xu et al., which uses biometric sensors, smart insoles, heart rate monitoring, and neural network analysis to provide real-time guidance to patients. This platform closes the feedback loop by automatically detecting exercise intensity and issuing alerts if deviations occur, without requiring direct human intervention. This approach enhances exercise safety and increases patient engagement, as shown by higher satisfaction scores and programme completion rates [24].

Beyond physiological guidance, AI has also been used to tailor behavioural and educational content, delivering personalised notifications, motivational messages, and dynamic recommendations based on the patient’s progress. Studies by Wu et al. and Xu et al. have demonstrated that automatically adapted interventions are perceived as more relevant and result in greater retention compared to standardised formats. Moreover, the reduced need for constant human interaction increases the feasibility of such interventions in resource-limited clinical environments.

**Figure 2:**
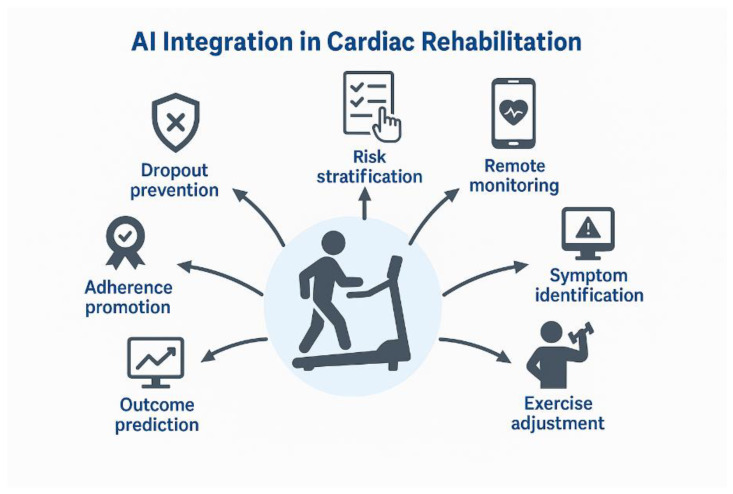
Artificial intelligence integration in cardiac telerehabilitation network.

Figure 2 conceptualises AI integration as a multidirectional clinical architecture rather than a single functional upgrade. The diagram illustrates how AI operates simultaneously across several domains, including dynamic risk stratification, exercise adjustment, dropout prediction, symptom identification, adherence reinforcement, and remote physiological monitoring. These components form a closed-loop adaptive system in which patient-generated data are continuously analysed and translated into real-time therapeutic modifications. This multidimensional integration differentiates AI-enhanced telerehabilitation from conventional digital platforms, which typically rely on linear, protocol-based workflows.

Integrating AI into telerehabilitation also helps reduce the burden on healthcare systems, as shown in Figure 2. Instead of requiring every patient to be manually monitored by a clinician, AI algorithms can analyse continuous data streams and flag only clinically significant deviations. This was evident in studies using intelligent alert systems, where AI identified anomalies in patient parameters and triggered either automated responses or targeted clinician alerts. As a result, the intervention’s effectiveness is maintained, and it also becomes scalable and sustainable within broader cardiovascular prevention programmes [24,25].

C. AI vs. Telerehabilitation: A Transformational Contrast

Comparing non-AI interventions with AI-driven ones underscores the need to move from passive, reactive digital solutions to proactive, intelligent systems. Traditional interventions, such as Corrie/MiCORE, LifePod, Cardioplan, and TeleCaRe, rely on standard protocols, automated reminders, and in some cases, physiological telemonitoring with manual interpretation. Although they provide functional support, they cannot respond to individual patient changes in real time [15,18,26,27].

In contrast, AI-driven systems provide a fundamentally different model in which collected data are not only monitored but also analysed, interpreted, and converted into real-time actions. In the study by Xu et al., patients were not merely informed that they had failed to reach their target heart rate zone—they were instantly alerted, and the algorithm adapted the exercise session accordingly. This real-time reactivity facilitates an optimisation of intervention that is simply not achievable with static telerehabilitation systems [24].

One key characteristic of AI systems is their capacity for predictive modelling and dynamic risk stratification. Unlike non-AI interventions, which operate linearly and cannot anticipate clinical events or patient dropout, AI models can estimate the risk of reinfarction, rehospitalization, or functional decline and adjust intervention frequency and resource allocation on a personalized basis. This distinction elevates AI from a supporting tool to a clinical decision-making agent.

A major limitation of non-AI platforms is that, despite incorporating modern features such as wearables, mobile applications, and automated feedback, they still require constant human oversight. For example, in the I-CREST programme, progress analysis was conducted weekly by a clinician, and adjustments were subject to delays. In contrast, AI interventions significantly reduce this “informational latency”, responding in real time with adaptive, scalable, and precise feedback. In overburdened healthcare systems, this becomes essential for long-term sustainability [28].

Furthermore, non-AI models are based on generalised, one-size-fits-all logics, with limited adaptation to the patient’s psychosocial context, risk profile, or lifestyle. This shortcoming has been highlighted in studies by Duscha et al., Maddison et al., and Claes et al., which, although showing positive effects on VO_2_ max, failed to demonstrate sustained improvements in daily physical activity. In contrast, AI-driven platforms can learn from patient behaviour and tailor not only physical training but also communication, education, and motivational strategies.

**Figure 3:**
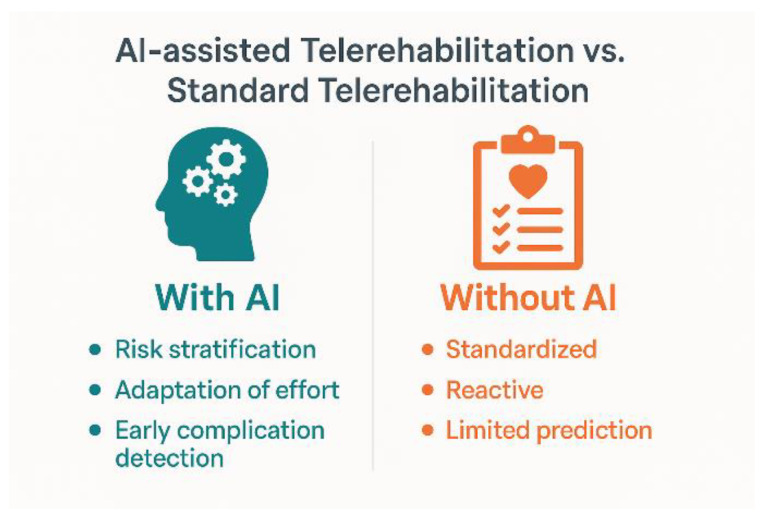
Telerehabilitation programmes with AI versus Standard telerehabilitation after ACS.

Figure 3 provides a conceptual comparison between conventional and AI-assisted telerehabilitation models. Rather than implying definitive clinical superiority, the diagram illustrates structural differences in system functionality. Standard telerehabilitation is typically protocol-driven and reactive, relying on periodic clinician review and fixed programming logic. In contrast, AI-assisted models incorporate predictive analytics, adaptive effort modulation, and early anomaly detection mechanisms. These differences reflect emerging architectural capabilities rather than established outcome superiority, as robust comparative randomized evidence remains limited.

The contrast between static and AI-powered systems is not merely technological—it is conceptual, suggested in Figure 3. AI-assisted telerehabilitation is not just about digitalisation but about autonomy, adaptability, and prediction. By integrating machine learning, behavioural and biometric analysis, and real-time feedback, AI elevates cardiac rehabilitation from passive monitoring to intelligent intervention. This paradigm shift enables the transition from partially effective solutions to a sustainable clinical ecosystem that anticipates, adapts, and optimises care for each patient [22,29,30].

D. AI for Post-ACS Risk Stratification

One of the most promising applications of AI in telerehabilitation is its ability to stratify post-infarction risk. Instead of using a one-size-fits-all rehabilitation model, AI allows for tailored care pathways based on each patient’s clinical, behavioural, and biological profile. Recent studies, such as those by Kulkarni et al., Razavi et al., and Wu et al., have developed algorithms that estimate the risk of reinfarction, rehospitalisation, non-adherence, or failure to return to work, with performance metrics surpassing those of conventional clinical scoring systems [25,31,32].

For example, the model developed by Kong et al. applied logistic regression and machine learning techniques to estimate ischemic event recurrence, reporting an AUC of 0.921. Nevertheless, the absence of systematic external validation and comparison with established risk scores (e.g., GRACE, TIMI) limits interpretation of its added clinical value [31]. Similarly, Razavi et al. and Wu et al. employed neural networks and multivariable analyses to integrate VO_2_ peak, imaging data, and medical history, resulting in robust models for forecasting functional prognosis and likelihood of socio-professional reintegration. These applications show that AI can be a crucial tool in identifying vulnerable patients before overt clinical complications arise.

Such predictive insights can be embedded within AI-driven telerehabilitation platforms to adjust not only the content of the intervention but also the frequency of sessions, level of supervision, and type of feedback provided. High-risk patients can receive more intensive interventions and targeted psychological support, while low-risk individuals may follow a more autonomous pathway, optimising resource allocation. This level of personalised triage is unachievable through traditional telerehabilitation platforms, which typically apply a uniform protocol across all patients, overlooking their complexity [25,32].

Furthermore, AI enables dynamic risk stratification, updating assessments based on a patient’s response to the intervention. For example, a patient initially classified as moderate risk who begins to exhibit signs of functional decline or poor adherence can be reclassified in real time, with the therapeutic trajectory adjusted accordingly. This functionality was demonstrated in simulations conducted by Wu et al. and Xu et al., in which AI systems detected early signs of dropout or decompensation and triggered automated response mechanisms. In this way, AI assesses risk and orchestrates an adaptive therapeutic response [24,25].

Although several AI-based models report high discriminatory performance (e.g., AUC > 0.9), direct prospective comparisons with established clinical risk scores such as GRACE or TIMI are rarely performed. Moreover, calibration metrics, net reclassification improvement (NRI), and decision-curve analyses are inconsistently reported across studies. As a result, while AI models demonstrate strong statistical discrimination, their incremental clinical utility over validated risk stratification tools remains insufficiently established. Future research should prioritise head-to-head comparisons and comprehensive model validation, including calibration and reclassification analyses, before clinical integration.

E. AI for the Personalization of Physical Effort

Personalising physical effort is essential to effective cardiac rehabilitation, but it is challenging to achieve without advanced technological support. Traditional methods determine exercise intensity at the start of a programme through baseline stress testing and periodic reassessments, which do not account for daily fluctuations in a patient’s physiological status. AI allows for a shift from this static approach to a dynamic model, where exercise intensity, duration, and frequency can be continuously adjusted based on the patient’s real-time physiological state, enabling precise, safe, and adaptive intervention.

The study by Xu et al. introduces an innovative dimension by using smart insoles embedded with pressure sensors and accelerometers that transmit real-time biomechanical data to a neural network. This system analyses gait patterns and posture to detect suboptimal effort or balance-related risks, providing real-time auditory and visual cues to the patient and adjusting exercise type and intensity autonomously, without requiring external input. This establishes a self-correcting, precise, and scalable training system.

This automated adaptation reduces clinical risks such as exercise-induced hypotension, fatigue, or arrhythmias and alleviates patient anxiety by ensuring their physical activity is intelligently monitored. At the same time, it reduces the burden on medical staff, who no longer need to monitor individual exercise sessions constantly. Compared to manual, clinician-led approaches, this AI-guided model offers a high degree of patient autonomy without compromising the safety or efficacy of the intervention [24].

F. AI as a Facilitator of Sustainability and Scalability

One of the main benefits of incorporating AI into telerehabilitation is its ability to make these interventions more scalable and sustainable. Cardiac rehabilitation relies heavily on human resources, including doctors, physiotherapists, nurses, and administrative staff, which limits its widespread adoption, especially in rural areas or underfunded healthcare systems. By automating tasks such as monitoring, data analysis, and programme adjustments, AI reduces the workload on medical teams and allows rehabilitation without continuous supervision.

Studies that implement AI components have demonstrated that many traditional tasks can be safely delegated to algorithms without compromising efficacy or patient safety. For example, in the IERMS system described by Xu et al., effort analysis and exercise session personalisation are fully automated, eliminating the need for permanent human oversight.

Scalability also involves maintaining quality as volume increases. AI offers a framework for simultaneously replicating the same standard of care across hundreds or even thousands of patients, regardless of their location. This enables the implementation of standardised telerehabilitation models without compromising personalisation—an essential aspect for healthcare systems dealing with growing numbers of post-ACS patients and limited resources [24].

Lastly, economic sustainability is a significant advantage of AI. By cutting personnel costs, enhancing adherence, and preventing complications through early intervention, AI substantially contributes to reducing the overall cost of rehabilitation. Pilot studies indicate that such platforms can be cost-effective, especially when integrated into existing e-health infrastructure. In this way, AI optimises care at the individual level and provides a viable and efficient solution for increasing access to cardiac rehabilitation at a population level.

G. AI in Early Diagnosis During the Subacute Post-ACS Window (0–14 Days)

One crucial but often overlooked stage in managing patients after a heart attack is the initial period after hospital discharge, especially the first 14 days following an ACS. During this fragile time, patients are more likely to experience reinfarction, irregular heart rhythms, worsening chest pain, or not sticking to their treatment. However, these complications often go unnoticed without ongoing monitoring until serious health problems arise. Traditional digital interventions without AI capabilities are usually limited to educational content or reminders and cannot detect early signs of instability.

It is important to distinguish between AI systems designed to adapt telerehabilitation interventions and AI tools developed for acute diagnostic triage. The studies discussed in this section primarily address AI-assisted ECG interpretation and early detection of ischemic events. While these technologies do not constitute telerehabilitation interventions per se, they may serve as complementary safety layers during the vulnerable subacute post-discharge phase, particularly when integrated into remote monitoring ecosystems.

AI has the potential to bridge this gap by acting as a predictive and interventional safety layer during the subacute phase. Recent studies confirm AI’s potential in supporting early clinical decision-making during this period. Within the ARISE project, deep learning algorithms were trained to interpret standard ECGs to distinguish between STEMI, NSTEMI, and unstable angina, achieving accuracy levels superior to those of junior physicians [33]. Another notable example is the use of AI to interpret prehospital ECGs (e.g., in ambulances), achieving over 90% sensitivity for STEMI detection [34]. Chen et al. developed a CNN-LSTM model capable of predicting silent STEMI in haemodynamically unstable patients by detecting subtle pre-infarction electrical patterns with high accuracy [35].

Moreover, these tools can be combined with data from wearable devices—such as ECG patches or smart wearables—to facilitate continuous out-of-hospital monitoring. AI algorithms can notify both the patient and the healthcare provider in real time, allowing for interventions before the onset of severe symptoms. This method is particularly effective for detecting silent ischemia, nocturnal angina, or paroxysmal arrhythmias. Thus, AI not only complements clinical care but also redefines the scope of telerehabilitation, transforming it from a passive recovery support tool into an active clinical instrument for reinfarction prevention during the subacute phase.

H. Quality of Life and Mental Well-Being

One of the key objectives of post-infarction cardiac rehabilitation, beyond physical recovery, is to improve quality of life and the patient’s mental well-being. Several studies have shown that depression, anxiety, and social isolation are common during the ACS period and may hinder both adherence and long-term prognosis. Traditional telerehabilitation provides limited educational and behavioural support, but such interventions remain superficial without continuous adaptation.

AI-assisted interventions introduce a new dimension—namely, the ability to personalise and sustain emotional support through adaptive messaging and dynamic feedback. A study by Pelly et al. demonstrated that users of an AI-enabled platform providing personalised motivational feedback reported significantly reduced anxiety and an improved sense of control over their illness [36].

Additionally, AI can detect early signs of psychological decline, such as reduced frequency of physical activity, diminished engagement with the digital platform, or changes in sleep patterns. In a study by Yang et al., the integration of machine learning algorithms enabled early identification of depressive symptoms and prediction of nonadherence. These signals were used to trigger automated alerts and interventions (e.g., calls, tailored messages), resulting in improved quality-of-life scores (measured via the SF-36) and a reduced risk of programme dropout [37].

Compared to static telerehabilitation, AI-driven systems provide continuous, contextualised psychological support without the need for constant involvement of medical personnel.

Simultaneously, several studies have documented the positive impact of AI on patient autonomy and socio-professional reintegration. For instance, the MiCORE platform, even without advanced AI, showed that patients receiving continuous digital support felt more confident in managing their condition [15]. The expansion of such platforms through AI integration, as seen in the studies by Wu and Xu, amplifies this effect by delivering recommendations that enhance perceived safety, reduce fear of exertion, and boost confidence in self-management abilities. In this way, AI contributes to physical recovery and directly to restoring a balanced, active, and psychologically resilient life following myocardial infarction [24,25].

I. Smart Devices and Robots in AI-Driven Telerehabilitation

At the heart of modern telerehabilitation are smart devices, which allow for the continuous collection of physiological, biomechanical, and behavioural data (Table 1). These tools are most effective when combined with AI algorithms to interpret data in real time and initiate automated interventions. Recent studies have shown that devices like the AliveCor KardiaMobile can detect atrial fibrillation with clinical accuracy and transmit data directly to mobile apps for AI-based interpretation [8]. Similarly, the Eko Core, a digital stethoscope with embedded AI functionality, can analyse heart sounds during rehabilitation to predict early signs of congestion or valvular dysfunction [38].

Integrating these technologies into AI-driven platforms, such as those tested by Xu et al. and Wu et al., enables the closure of the physiological feedback loop. In this context, AI does not just collect data from wearables (e.g., heart rate, oxygen saturation, activity level); it also delivers personalised decisions, alerts the patient, autonomously adjusts the exercise regimen, or notifies the clinical team. Wearable devices like ECG patches, accelerometers, and smart insoles contribute to this AI-assisted clinical autonomy, with studies showing improved patient retention and safety during home-based rehabilitation [24,25].

A notable example of this integration is the NAO platform—an interactive social robot used experimentally to help patients with home exercises and deliver personalised motivational messages via AI. A pilot study by Pelly et al. reported a significant increase in patient engagement and satisfaction compared to traditional apps [36]. In parallel, the VA Smart Home Telerehabilitation System by Hilu et al. demonstrated that integrating smart cameras with motion-recognition algorithms enables precise monitoring of exercise quality performed at home [17].

Thus, when properly integrated with AI, smart devices become extensions of the healthcare system, rather than just passive data-recording instruments.

## 4. Discussion

The comparative synthesis presented in Table 2 provides a structured overview of the differential evidence base supporting conventional telerehabilitation and AI-enhanced telerehabilitation across key outcome domains, thereby contextualising the subsequent discussion within a balanced appraisal of established findings and emerging translational evidence.

Rather than reiterating individual study findings, this discussion aims to synthesise the collective evidence and interpret its implications for clinical implementation and future research. The contrast between established telerehabilitation models and emerging AI-enhanced systems reflects not only technological progression but also a shift in the conceptual framework of cardiac rehabilitation delivery.

Cardiac telerehabilitation has evolved significantly over the past decade, transitioning from pilot telehealth projects to structured mHealth platforms. Research studies, such as Claes et al., Avila et al., and Maddison et al., have shown that telerehabilitation can be just as effective as traditional centre-based programmes in improving functional parameters, such as VO_2_ peak, and patient satisfaction. However, these interventions remain fundamentally reactive, standardised, and reliant on regular clinician input [20,21,22,30]. Their long-term adherence, behavioural sustainability, and scalability limitations have become increasingly apparent. In contrast, integrating AI into telerehabilitation marks a shift from static digital care to intelligent, dynamic, and personalised interventions. AI enhances existing models by converting data into clinical action, enabling real-time adjustment of exercise intensity, continuous physiological monitoring, and early detection of risk trajectories [24,25].

Traditional telerehabilitation programmes struggle to tailor care beyond the initial assessment. AI fills this gap by continually analysing biometric, behavioural, and contextual data. The IERMS platform showcases this progress by using smart insoles, heart rate sensors, and deep neural networks to personalise and adjust exercise in real time without clinician intervention [24]. AI also improves behavioural feedback loops. Studies by Wu et al. and Pelly et al. demonstrate that AI-generated motivational messaging and adherence predictions can adapt content to the user’s behaviour, mood, and engagement level, significantly improving retention and clinical outcomes [25,36].

AI is uniquely positioned to provide dynamic risk stratification in post-ACS patients. Models developed by Kulkarni et al., Kong et al., and Razavi et al. have shown high predictive accuracy (AUC > 0.9) for outcomes such as reinfarction, hospital readmission, and VO2 decline. These tools enable real-time resource reallocation, escalation of care for high-risk individuals, and automation of clinical decision-making [31,32,39].

Moreover, during the subacute window (first 14 days post-ACS), AI has proven useful in early diagnosis and prevention. The ARISE initiative demonstrated AI’s superiority over junior physicians in interpreting ECGs for STEMI/NSTEMI differentiation [33,34]. CNN-LSTM models by Chen et al. have accurately detected silent ischemia and arrhythmias in ambulatory settings, opening the door to integrating predictive diagnostics into telerehabilitation workflows [35].

One long-standing limitation of telerehabilitation platforms has been their dependence on clinician supervision. AI tackles this issue by allowing closed-loop systems to self-regulate, provide intelligent alerts, and escalate care independently. The system developed by Xu et al. substantially reduced the need for ongoing human oversight while maintaining safety and effectiveness [24]. Scalability is further improved as AI enables the replication of complex interventions across large populations without compromising personalisation [15,25]. From an economic perspective, AI enhances sustainability. Programmes incorporating AI have demonstrated reduced resource usage, lower readmission rates, and improved adherence, making them cost-effective in real-world settings [24,40].

Aside from physical outcomes, AI has started to tackle the psychosocial aspects of recovery. Studies by Wu et al. and Yang et al. used behavioural algorithms to spot early signs of depression, anxiety, or motivational slumps. These systems prompted automatic interventions that boosted quality-of-life metrics (SF-12, EQ-5D) and helped people reintegrate socially [25,37].

The use of socially interactive robots and voice agents extends AI’s emotional support capabilities, creating empathetic digital interfaces that drive long-term behavioural change [36].

Conventional telerehabilitation is limited to reactive care, focusing on recording symptoms or metrics for review. AI allows telerehabilitation to evolve into a real-time clinical companion, anticipating deterioration, guiding safe exercise, and adapting to changing health conditions. This is particularly valuable for high-risk or remote populations.

As Kong et al. and Xu et al. demonstrated, intelligent stratification and early warning systems transform telerehabilitation from a monitoring platform into a proactive therapeutic ecosystem [24,39].

Although there is strong theoretical and pilot-phase evidence, several challenges persist. Most AI-integrated programmes are still in their early stages, single-centre, and lack RCT-level validation. Barriers to widespread adoption include interoperability with electronic health records, algorithm explainability, and minimising bias.

Patients’ and clinicians’ trust in AI systems also relies on transparency, data privacy assurances, and demonstrable clinical value. Acceptance studies, such as those by Pelly et al., highlight the importance of co-design and human-centred implementation.

Future research must prioritise large-scale trials comparing AI+telerehabilitation with standard CR, measure hard clinical endpoints (MACE, mortality), and establish cost–benefit frameworks across diverse healthcare settings [36].

AI-enhanced telerehabilitation marks a significant departure from traditional cardiovascular recovery. By automating personalisation, risk detection, and therapeutic feedback, AI expands cardiac rehabilitation’s scope, depth, and responsiveness. Its integration is no longer optional, but crucial for developing scalable, sustainable, and equitable models of post-ACS care. A future where rehabilitation can learn, adapt, and respond in real time is no longer a hypothetical prospect—it is already emerging, and AI is the driving force behind this transformation.

While AI-enhanced telerehabilitation offers promising adaptive capabilities, its implementation is not without potential risks. Algorithmic errors, misclassification of clinical deterioration, or false reassurance may delay necessary medical intervention. Predictive models trained on limited or unrepresentative datasets may introduce bias, disproportionately affecting older adults, socioeconomically disadvantaged populations, or patients with multimorbidity.

Over-reliance on automated systems may also reduce clinician vigilance, particularly if AI outputs are perceived as authoritative rather than probabilistic. In the rehabilitation context, incorrect exercise intensity modulation or failure to detect arrhythmogenic patterns could theoretically result in patient harm. Furthermore, issues related to data security, interoperability, and explainability remain unresolved in many early-phase systems.

For these reasons, AI-driven telerehabilitation should be implemented within a supervised clinical framework in which algorithms function as decision-support tools rather than autonomous decision-makers. Transparent validation, external benchmarking, real-world safety monitoring, and regulatory oversight are essential to ensure that innovation does not compromise patient safety.

The role of structured cardiac rehabilitation extends beyond post-ACS care. In atrial fibrillation (AF), recent literature emphasizes the importance of cardiorespiratory fitness and moderate-intensity exercise in reducing AF incidence and recurrence, as reflected in current European Society of Cardiology guidelines. Comprehensive rehabilitation programs in AF integrate prognostic stratification, pharmacological optimisation, comorbidity management, structured physical training, and psychosocial support. Such multidisciplinary frameworks demonstrate that rehabilitation is not merely adjunctive but integral to cardiovascular disease management. This broader perspective reinforces the rationale for integrating adaptive and AI-enhanced models into post-ACS rehabilitation, aligning digital innovation with established multidisciplinary rehabilitation principles [41].

The overall level of evidence supporting AI-enhanced telerehabilitation remains moderate to low, primarily due to the predominance of pilot trials, single-centre investigations, feasibility studies, and simulation-based analyses. While several studies report promising functional, behavioural, and predictive outcomes, the certainty of evidence varies across domains. Evidence is strongest for feasibility, adherence monitoring, and risk modelling, whereas long-term clinical outcomes, cost-effectiveness, and comparative superiority over standard cardiac rehabilitation remain insufficiently established. These considerations align conceptually with GRADE principles, highlighting the need for cautious interpretation and further high-quality randomized trials. While several AI-based models report strong discriminatory performance (e.g., AUC values exceeding 0.9), rigorous benchmarking against validated and widely implemented clinical risk scores such as GRACE or TIMI remains limited. Furthermore, calibration metrics, decision-curve analysis, and net reclassification improvement are inconsistently reported. Therefore, although AI-driven risk prediction appears promising, meaningful contextualisation of predictive performance requires direct comparative validation against established prognostic frameworks before clinical integration can be recommended.

This narrative review has several limitations that should be considered when interpreting its conclusions. Although the literature search was structured and covered multiple major databases, the review was not conducted as a fully systematic analysis with protocol registration or formal risk-of-bias assessment, which introduces potential selection and interpretation bias. In addition, there is marked heterogeneity among the included studies regarding telerehabilitation formats, artificial intelligence methods, and reported outcomes, limiting direct comparability and preventing quantitative synthesis. Furthermore, many AI-based predictive models report high discrimination metrics (AUC), yet lack consistent calibration analysis, comparison with validated clinical risk scores (e.g., GRACE, TIMI), or reclassification metrics, limiting conclusions regarding incremental clinical value.

The evidence base for AI-enhanced telerehabilitation is still developing, with most studies being pilot or single-centre investigations, small sample sizes, and short follow-up durations. Consequently, robust data on long-term effectiveness, sustainability of behavioural change, cost-effectiveness, and hard clinical endpoints such as major adverse cardiovascular events or mortality are scarce. Moreover, patient populations are often selectively recruited, favouring digitally literate and motivated individuals, which raises concerns about generalisability to older, socioeconomically disadvantaged, or multimorbid post-ACS patients.

The evidence landscape demonstrates asymmetry between conventional telerehabilitation and AI-enhanced approaches. While telerehabilitation is supported by multiple RCTs confirming feasibility and non-inferiority to centre-based rehabilitation, AI-integrated interventions remain largely represented by modelling studies, retrospective analyses, pilot trials, and early-stage feasibility protocols. Consequently, the overall certainty of evidence supporting AI-enhanced telerehabilitation is moderate to low, particularly for hard clinical endpoints such as major adverse cardiovascular events or mortality. Stronger evidence currently supports feasibility, predictive modelling accuracy, and adherence optimisation rather than definitive superiority over established rehabilitation paradigms.

AI may represent an important next step in the evolution of digital cardiovascular recovery, provided that its clinical benefits are confirmed through robust randomized controlled trials and real-world validation studies. Finally, important practical challenges remain insufficiently addressed, including limited coverage of the high-risk early post-discharge period, variable inclusion of the full ACS spectrum, and unresolved issues related to AI transparency, bias, data privacy, interoperability with electronic health records, and clinician trust. These factors currently constrain the widespread clinical implementation of AI-driven telerehabilitation despite its demonstrated potential.

Future research should prioritise pragmatic, multicentre trials evaluating AI-enhanced telerehabilitation within real-world cardiology workflows, including community hospitals and outpatient cardiac units. Implementation science approaches, clinician acceptance studies, and reimbursement modelling will be critical to determine feasibility beyond controlled research environments. Bridging the gap between algorithmic potential and routine cardiovascular care remains a central challenge for the next phase of development.

## 5. Conclusions

Cardiac telerehabilitation has demonstrated feasibility and moderate effectiveness in improving functional and behavioural outcomes after ACS. Nevertheless, most currently implemented digital programmes remain largely standardised, reactive, and dependent on continuous human supervision, which limits their scalability, long-term engagement, and capacity for individualised risk management.

The integration of AI introduces a qualitative shift in this landscape by enabling automated personalisation, dynamic risk stratification, and real-time adaptive responses. Emerging evidence from AI-driven platforms, including those described by Xu et al. [24], Wu et al. [25], and Kong et al. [39], suggests that intelligent systems can safely adjust exercise intensity, anticipate functional or behavioural decline, enhance adherence, and reduce rehospitalisation risk. Importantly, these capabilities extend beyond traditional telerehabilitation models and address several structural limitations of both centre-based and conventional remote cardiac rehabilitation.

Beyond physical recovery, AI-enhanced telerehabilitation shows potential in supporting sustained behavioural change, identifying psychosocial vulnerabilities, and fostering patient autonomy through continuous, context-aware feedback. By integrating physiological, behavioural, and contextual data, AI enables rehabilitation pathways that are not only remote but adaptive and proactive.

Despite these advances, the field remains at an early stage of clinical translation. Most available studies are pilot or feasibility trials with limited sample sizes and short follow-up periods. Robust evidence from large-scale, multicentre randomized controlled trials, cost-effectiveness analyses, and real-world implementation studies is still required to confirm long-term clinical benefits, safety, and health-system impact.

In conclusion, the convergence of artificial intelligence and telerehabilitation may represent an important evolutionary step in post-ACS care. Current evidence suggests potential benefits in adaptive monitoring, personalised intervention, and predictive risk modelling; however, these findings are predominantly derived from pilot and early-phase studies. The field remains in a translational stage, with substantial areas of uncertainty and ongoing methodological refinement. Rather than constituting a definitive clinical transformation, AI-enhanced telerehabilitation should be regarded as a promising hypothesis that warrants rigorous validation through large-scale, multicentre trials and real-world implementation studies.

## Figures and Tables

**Table 1:** Studies that include AI in cardiac rehabilitation programmes.

Study	Type of Intervention	AI Function	Primary Outcome of AI	AI Model	AI Time Using	Scalability
Xu et al. (2020) [24]	AI + Telerehab	Smart insoles, dynamic stratification	Increased safety and adherence	Smart insoles + NN	6 weeks	Medium
Wu et al. (2025) [25]	AI + Telerehab	Prediction, personalized feedback	Reduced readmission, improved quality of life	Hybrid AI behavioral + physio	12 weeks	High
Kulkarni et al. (2021) [31]	AI	Functional prognostic prediction	High accuracy in VO_2_ estimation	Machine learning (ML)	12 weeks	High
Razavi et al. (2025) [32]	AI	Deep Learning model for prediction	Effective risk stratification	Deep learning (DL)	Variable	Medium-High
Zhang et al. (2023) [38]	AI	Scar analyses on CMR	Detection of myocardial scar	CNN, deep learning	1 time	Limited
Kong et al. (2022) [39]	AI	New-onset of ACS prediction	AUC > 0.92	Logistic regression (LR), ML	NA	High

**Table 2:** Comparative Summary of Telerehabilitation vs. AI-Enhanced Telerehabilitation in Post-ACS Care.

Outcome Domain	Conventional Telerehabilitation	AI + Telerehabilitation	Evidence Level
Functional capacity (VO_2_ peak)	Demonstrated non-inferiority to centre-based CR in multiple RCTs	Limited interventional data; indirect support via adaptive intensity modulation	High (Telerehab)/Low–Moderate (AI)
Medication adherence	Improved via reminders and behavioural messaging	Enhanced through adaptive, personalised feedback and dropout prediction	Moderate (Telerehab)/Low (AI)
Dynamic risk stratification	Static baseline assessment; limited real-time adaptation	Predictive modelling for reinfarction, readmission, and functional decline	Low (AI modelling; limited RCT validation)
Exercise intensity modulation	Predefined protocols with periodic clinician adjustment	Real-time adjustment using biometric feedback and ML algorithms	Low–Moderate (Pilot-level evidence)
Psychosocial monitoring	Periodic assessment; limited adaptive feedback	Behavioural pattern recognition; early detection of disengagement	Low (Feasibility studies)
Scalability	Requires clinician oversight; moderate scalability	Automation reduces clinician burden; high theoretical scalability	Moderate (conceptual; limited RCT)
Early post-discharge monitoring (0–14 days)	Limited monitoring capability	AI-enabled early warning systems (diagnostic adjuncts)	Low (diagnostic validation; not rehab RCT)
Hard clinical endpoints (MACE, mortality)	Limited but supported by longer-term CR evidence	Insufficient data from large RCTs	Moderate (Telerehab)/Very Low (AI)

## Data Availability

No new data were created or analyzed in this study. Data sharing does not apply to this article.
